# Dynamic Characterization of the Biomechanical Behaviour of Bovine Ovarian Cortical Tissue and Its Short-Term Effect on Ovarian Tissue and Follicles

**DOI:** 10.3390/ma13173759

**Published:** 2020-08-25

**Authors:** Giulia Pascoletti, Maddalena Di Nardo, Gionata Fragomeni, Vincenza Barbato, Teresa Capriglione, Roberto Gualtieri, Riccardo Talevi, Gerardo Catapano, Elisabetta M. Zanetti

**Affiliations:** 1 Department of Engineering, University of Perugia, 06125 Perugia, Italy; giulia.pascoletti@studenti.unipg.it; 2 Department of Biology, University of Naples Federico II, 80100 Naples, Italy; dinardomaddalena@gmail.com (M.D.N.); barbato_vincenza@libero.it (V.B.); teresa.capriglione@unina.it (T.C.); roberto.gualtieri@unina.it (R.G.); riccardo.talevi@unina.it (R.T.); 3 Department of Medical and Surgical Sciences, Magna Graecia University, 88100 Catanzaro, Italy; fragomeni@unicz.it; 4 Department of Mechanical, Energy and Management Engineering, University of Calabria, 87030 Rende (CS), Italy; gerardo.catapano@unical.it

**Keywords:** ovarian tissue, creep, elastic modulus, tensile test, viscous behavior, biomechanics

## Abstract

The ovary is a dynamic mechanoresponsive organ. In vitro, tissue biomechanics was reported to affect follicle activation mainly through the Hippo pathway. Only recently, ovary responsiveness to mechanical signals was exploited for reproductive purposes. Unfortunately, poor characterization of ovarian cortex biomechanics and of the mechanical challenge hampers reproducible and effective treatments, and prevention of tissue damages. In this study the biomechanical response of ovarian cortical tissue from abattoir bovines was characterized for the first time. Ovarian cortical tissue fragments were subjected to uniaxial dynamic testing at frequencies up to 30 Hz, and at increasing average stresses. Tissue structure prior to and after testing was characterized by histology, with established fixation and staining protocols, to assess follicle quality and stage. Tissue properties largely varied with the donor. Bovine ovarian cortical tissue consistently exhibited a nonlinear viscoelastic behavior, with dominant elastic characteristics, in the low range of other reproductive tissues, and significant creep. Strain rate was independent of the applied stress. Histological analysis prior to and after mechanical tests showed that the short-term dynamic mechanical test used for the study did not cause significant tissue tear, nor follicle expulsion or cell damage.

## 1. Introduction

Female infertility has been ranked by the World Health Organization (WHO) as the fifth most serious global cause of disability [1]. In women with primary ovarian insufficiency (POI), or ovarian tumor, the disease or the therapeutic treatment may destroy the entire natural follicle reserve, with which the patient was born. Timely cryopreservation and orthotopic transplantation of ovarian tissue is an effective approach in preserving fertility of these patients, and in making it possible for them to give birth to a baby [2]. The possible transmission of tumor cells on autotransplantation limits the use of such an approach for patients with blood-borne or metastatic tumors [3,4,5]. A safer alternative is to isolate and transplant only the patient’s follicles, either at the immature primordial stage or after a two-step in vitro culture. In fact, the follicle basal lamina prevents tumor cells from invading oocytes and follicular cells. In the two-step approach primordial follicles are first cultured inside fragments of ovarian tissue (i.e., in situ) till they reach the secondary stage, then they are isolated and cultured in vitro till full maturation [6,7,8,9,10]. The in situ culture step permits exploiting the complex array of dynamic biochemical and mechanical signals provided by the cells, and the extracellular matrix of the cortical tissue, to promote primordial follicle growth. Its efficiency in promoting follicle activation and maturation to ovulation has been shown in the mouse model [9], but was poor in humans [11]. In the last few years, the need to improve the poor long-term viability and growth of primordial follicles to the secondary stage in situ has drawn attention and research efforts to the optimization of media and bioreactor designs, for the in vitro culture of fragments of ovarian cortical tissue from large mammals [12]. Recently, experimental and theoretical evidence has been provided suggesting that ensuring an optimal oxygen availability in tissue may also help exploit tissue capacity to sustain follicle growth [13,14].

Biomechanical cues contribute to regulating development and homeostasis of many tissues together with biochemical cues. It has been shown that, challenging mechano-responsive cell constructs with mechanical signals during culture, in mechanical bioreactors, may guide cell morphology, proliferation, and differentiation, as well as the organization and functions of multiple cell types in engineered tissues such as bone, ligament, tendon, and muscle [15].

The ovary is a dynamic mechano-responsive organ with hierarchical structural organization, as nicely reviewed in [16]. Collagen concentration in the stroma decreases from the outer cortical to the inner medullary region, causing a corresponding decrease of tissue stiffness. Primordial follicles are generally present in the outermost stiff cortex. When they are recruited for reproduction, follicles grow and move towards the softer medulla along their maturation pathway to the pre-antral and antral stage. Follicles in stiffer alginate matrices have been shown to secrete higher amounts of androgen and progesterone and lower amounts of estrogen, and become responsive to hormones as they move towards the medulla [17]. The mechanical forces acting on ovarian tissue trigger two main mechano-transduction pathways, the Hippo and the Akt (protein kinase B) pathways, that are potent regulators of primordial follicle activation. In particular, alteration of the mechanical forces acting on tissue (e.g., by tissue fragmentation) disrupts the Hippo pathway and promotes primordial follicle activation [18]. Ovulation (i.e., the expulsion of the oocyte from the dominant follicle) occurring at the end of the maturation process is also dependent on mechanical signals, such as the stiffness of the extracellular matrix (ECM) and the increase of the intrafollicular pressure.

The potential for the applicative purpose of ovarian tissue response to mechanical challenges is generally underestimated. Only recently, two approaches have been proposed to exploit the mechanoresponsive behavior of ovarian tissue to enhance the effectiveness of cortical tissue, or follicle autotransplantation, in premature ovarian insufficiency patients. In the first approach, quiescent follicles are activated by the in vitro culture of mechanically disrupted fragments of ovarian tissue in the presence of biochemicals, promoting activation of the Akt signaling pathway. Tissue transplantation in POI patients has been reported to lead to successful pregnancies [19]. In the second approach, the number of viable and healthy follicles growing in fragments of ovarian cortical tissue in situ has been increased by stretching the fragments along one dimension by at least 10% of the initial size prior to the in vitro culture [20]. It is speculated that mechanical stretching loosens up and decreases density of the collagen fibers in the stroma, and increases the average distance among the primordial follicles, which would facilitate protein and cell movement in tissue and would remove the factors inhibiting follicle activation. One problem is that, the biomechanics of ovarian tissue is still poorly characterized. For this reason, it is very difficult to define in quantitative terms the mechanical challenge that effectively promotes follicle activation without causing damage to tissue, such as tissue tear, follicle expulsion, or rupture of ovarian cells followed by the release of their content.

To overcome such a gap, in this preliminary study we characterized the biomechanical response of fragments of fresh ovarian cortical tissue to a short-term dynamic tensile challenge and its possible damages to tissue. Bovine tissue was used as a large animal model of human tissue behavior.

## 2. Materials and Methods

### 2.1. Materials

**Figure 1:**
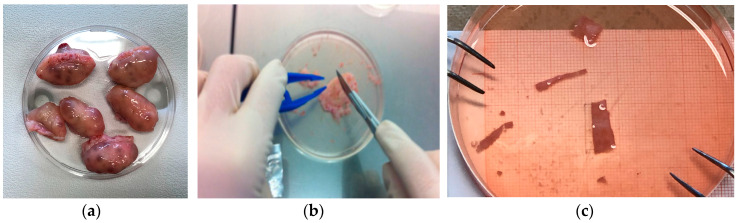
Specimens preparation: (**a**) fresh bovine ovaries; (**b**) fragment dissection; (**c**) tissue fragments.

Twenty-eight fresh ovaries were harvested from abattoir bovines younger than 2 years at a local slaughterhouse (Ponte S. Giovanni, Italy), and were transported to the laboratory within 2 h from slaughter in Leibovitz’s L-15, 1% penicillin-streptomycin, 1 µg/mL amphotericin-B, at 4 °C. Ovaries were immersed in Leibovitz’s L-15, 2 mM glutamine, 3 mg/mL BSA, 1% penicillin-streptomycin, 1 µg/mL amphotericin B solution (hereafter referred to as handling medium), and fragments of ovarian cortical tissue about 1 mm thick, 5 mm wide, and 15 mm long were dissected with a scalpel, as shown in Figure 1. The actual size of exemplary fragments was verified by mechanical and histological means. Tissue was only harvested from smooth and regular regions of the ovary which presumably had never ovulated. Leibovitz’s L-15 medium was purchased from Invitrogen (Milan, Italy). Penicillin streptomycin 100X, amphotericin B 250 µg/mL, bovine serum albumin (BSA), L-glutamine 200 mM, and eosin-Y were purchased from Sigma Aldrich (Milan, Italy). Mayer’s hematoxylin and paraffin wax were purchased from Carlo Erba (Milan, Italy).

### 2.2. Mechanical Characterization

**Figure 2:**
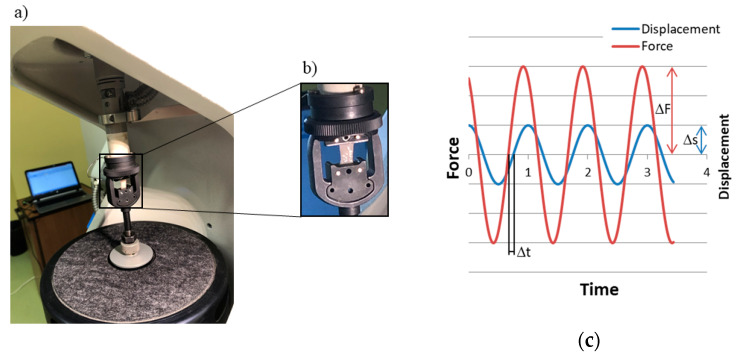
Mechanical testing: (**a**) the test machine; (**b**) a fragment mounted between clamps; (**c**) exemplary force and displacement recording.

The biomechanical behavior of ovarian cortical tissue was characterized with an approach similar to that used for dermal tissue in a previous study [21]. Briefly, the tissue fragments were subjected to uniaxial tensile dynamic testing with a DMA/SDTA861e (Mettler-Toledo GmbH, Schwarzenbach, Switzerland) (Figure 2a,b) at a mean stress amplitude *σ_a_* equal to 2.5 kPa and at varying average stress levels *σ_m_*, i.e., 150%, 300%, 500%, with respect to the dynamic stress amplitude. The initial clamp-to-clamp distance was equal to 10.5 mm. The test frequency was increased from 12 to 30 Hz, at 1 Hz steps. Tissue fragments were kept wet during the whole test procedure.

The mechanical properties of the tissue undergoing the harmonic loading were expressed in terms of its complex modulus [22]. The amplitude of the complex modulus (*E_c_*) was estimated from the ratio between the force and the displacement amplitude (Figure 2c) for the given fragment geometry, as follows:

(1) $$\left|E_{c}\right|=\frac{\Delta F}{\Delta s}\cdot \frac{l_{o}}{A}$$

where Δ*F* is the applied force amplitude, Δ*s* is the measured displacement amplitude, *l_0_* is the initial clamp-to-clamp distance, and *A* is the initial specimen cross-sectional area. The phase of the complex modulus was obtained experimentally as the phase angle *δ* between the load and the respective displacement signal (Figure 2c).

Tissue capacity to store energy reversibly (a measure of tissue elastic behavior) was characterized in terms of its elastic (or storage) modulus *E_e_*, as follows:

(2) $$E_{e}=\left|E_{c}\right|\cdot \cos\delta$$

As reported below, the tissue displayed non-linear elastic behavior with a current elastic modulus changing at different strain amplitudes. For this reason, unless otherwise noted, *E_e_* was averaged over the stress range from *σ_m_ − σ_a_ to σ_m_ + σ_a_*.

The mechanical energy irreversibly wasted to heat (a measure of tissue viscoelastic behavior) was characterized in terms of its loss modulus, *E_l_*, as follows:

(3) $$E_{l}=\left|E_{c}\right|\cdot \sin\delta$$

The relative importance of the viscous to the elastic tissue response was characterized in terms of the loss tangent tan *δ*, as follows:

(4) $$\tan\;\delta =\frac{E_{l}}{E_{e}}$$

Tissue creep was characterized by recording in time the strain (i.e., the displacement-to-initial length ratio, Δ*s/l_0_*) required to maintain the applied force constant. Strain vs. time curves were obtained at varying applied forces (i.e., stress). Experimental data were described in terms of a Newtonian dashpot of viscosity *η.* The model predicts that at a constant applied stress *σ_m_* tissue deformation *ε(t)* changes in time as follows [22]:

(5) $$\epsilon =\epsilon_{0}+\frac{\sigma_{m}}{\eta \;}t$$

The creep strain rate was estimated as the slope of the regression line through the experimental points in the secondary steady linear increase of tissue deformation over time.

### 2.3. Histologic Characterization

Tissue structure prior to and after mechanical testing was characterized by histological analysis, as follows. Cortical fragments were dissected at 4 °C in handling medium, fixed in Bouin’s solution, dehydrated in solutions at increasing ethanol concentrations, and embedded in paraffin. In the next step, 5 μm thick serial sections were obtained with a microtome and were stained with hematoxylin and eosin. Follicles were observed under an optical microscope by two experienced observers, and their quality and stage was assessed as follows. Grading of follicle quality [23]: grade 1 follicles are spherical with homogeneously distributed granulosa cells (GCs) and oocyte presenting a homogenous cytoplasm; grade 2 follicles have GCs non-uniformly distributed around the spherical oocyte; grade 3 follicles have pyknotic GCs and distorted and/or vacuolized oocyte. Staging of follicle growth [24]: primordial follicles have a complete layer of flat GCs or a layer composed of flat and cuboidal GCs; primary follicles have one complete layer of cuboidal GCs; secondary follicles have two or more complete layers of cuboidal GCs.

### 2.4. Statistical Analysis

Data were generally reported as mean +/− standard deviation. The Lilliefors test for normality was carried out to assess that experimental data were normally distributed. For normally distributed data, the 2-way ANOVA was used to assess the influence of frequency and average applied stress on tissue elastic and viscous properties. When data distribution significantly differed from a normal curve, the non-parametric Friedman test was used to test significance of differences. The influence of the applied stress on the strain rate in creep experiments was assessed with the Kruskal-Wallis test. The statistical significance of grading and staging differences was assessed with Fisher’s exact test for pairwise comparisons. A significance level of 0.05 was used throughout.

## 3. Results

### 3.1. Mechanical Characterization

**Figure 3:**
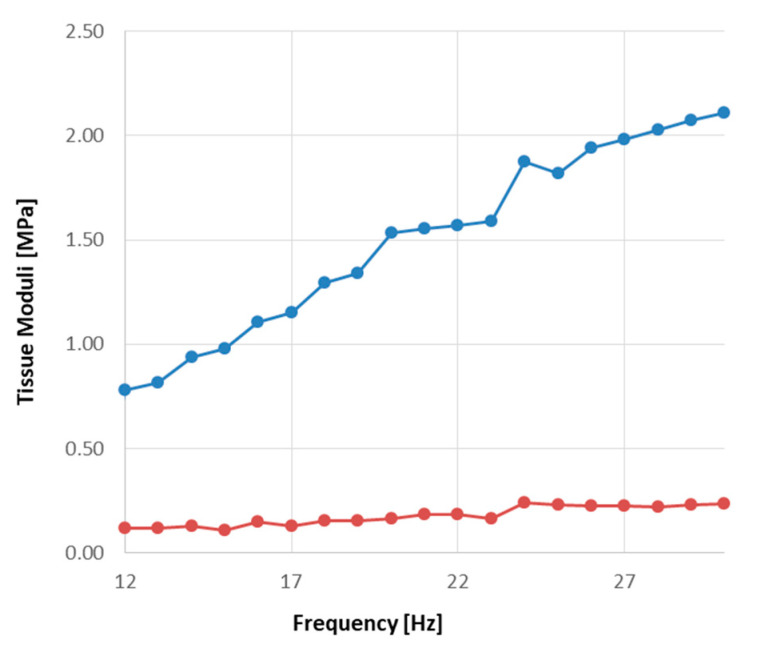
Dependence on frequency of the current elastic, *E_e_*, and loss modulus, *E_l_*, of an exemplary fragment of bovine ovarian tissue: (**•**,—) *E_e_*; (**•**,—) *E_l_*_._

Generally, ovarian tissue exhibited a viscoelastic behavior, as the parameters reported in Figure 3 show for an exemplary fragment. At low frequencies, the storage modulus *E_e_* increased with increasing frequency and approached an asymptote at high test frequencies. The loss modulus, *E_l_*, was consistently lower than *E_e_*, by as much as an order of magnitude and varied little with the test frequency. The loss tangent was consistently tan δ < 1, suggesting that tissue response under the investigated conditions was mainly elastic.

**Figure 4:**
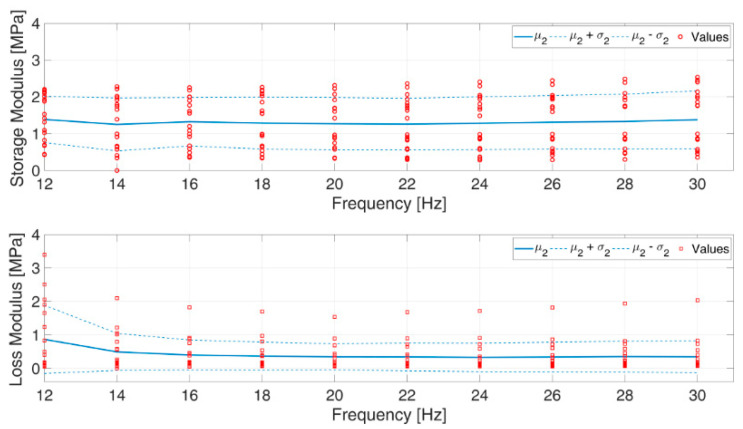
Spread of elastic, *E_e_*, and loss modulus, *E_l_*, data of bovine tissue from various ovaries.

The mechanical characteristics of ovarian tissue varied from ovary to ovary by slightly less than an order of magnitude, and concealed to some extent the intrinsic mechanical behavior of tissue from any given ovary, as shown in Figure 4. Figure 1 shows that to satisfy the size required of specimens for the mechanical characterization, each tissue fragment took up a large surface of the entire ovary. This prevented replicating the biomechanical test on multiple specimens of a given ovary, and investigating possible effects of the place from which the fragment was excised.

**Figure 5:**
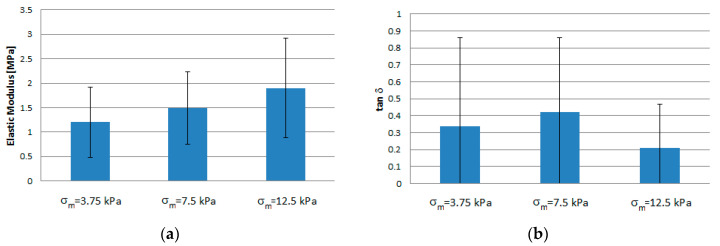
Elastic and viscous properties of ovarian tissue at different average stress *σ_m_*: (**a**) storage modulus (*E_e_*); (**b**) loss tangent (*tan δ*).

*E_e_* data were generally normally distributed at all frequencies, whereas tan δ data significantly deviated from a normal distribution (*p* < 3 · 10^−3^). As an effect of the large spread of data, neither the storage modulus *E*_e_, nor *tan δ* were significantly affected by the test frequency (Figure 4). Table 1 and Figure 5 show that the values of *E_e,_* averaged over the test frequencies, slightly increased with increasing average applied stress, whereas the frequency-averaged *tan δ* peaked at the intermediate average stress. Both *E_e_* (*p* < 10^−5^) and *tan δ* (*p* < 3 · 10^−4^) were significantly influenced by the average applied stress *σ_m_*.

**Figure 6:**
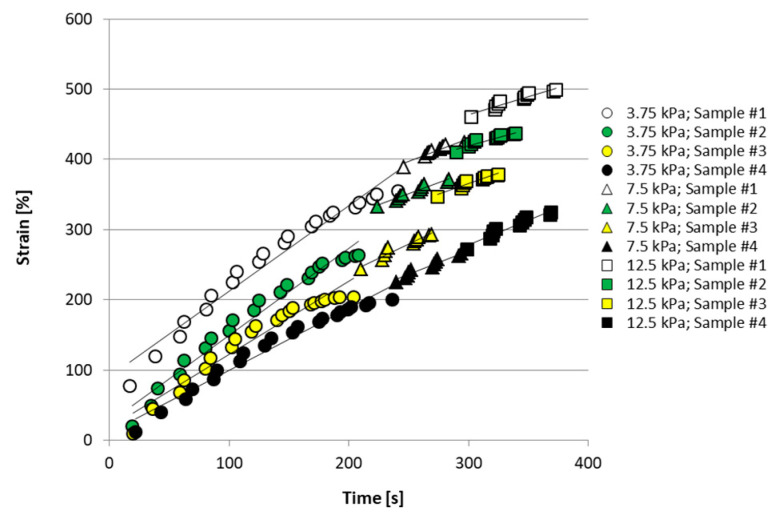
Strain vs. time curves in creep experiments on four exemplary fragments of bovine ovarian cortical tissue, at increasing average applied stress. Predictions of the viscous model are reported as solid lines. For details, please see text.

The creep tests confirmed that ovarian tissue exhibits viscous behavior on a long-time scale (i.e., beyond 50 s), as shown in Figure 6. Tissue primary deformation increased linearly and rapidly in time after application of the external stress, but then it slowed down attaining a secondary steady linear increase. At any given time, the occurring deformation significantly varied with the donor ovary, by up to a factor of four.

Figure 6 shows, that for exemplary tissue fragments, the viscous model described, fairly well, tissue response to creep tests. Interestingly, the strain rate did not significantly vary, either with the tissue source, or with the average applied stress, featuring an average of 1.10·10^−4^ s^−1^ (Table 1). Application of the highest investigated stress caused creep rupture of one tissue sample.

### 3.2. Tissue Characterization

**Figure 7:**
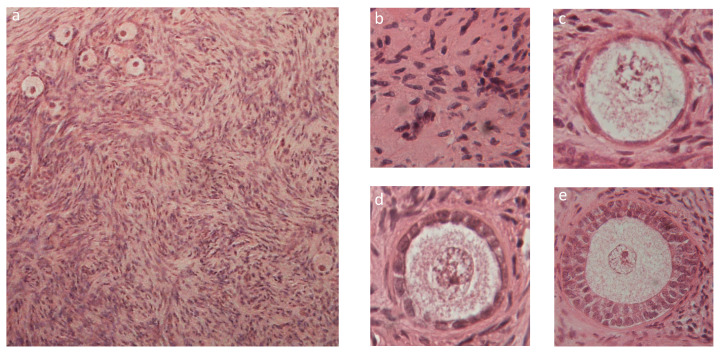
Exemplary histological section of cortical ovarian tissue showing its cellular components: (**a**) whole section; (**b**) stromal cells; (**c**) primordial follicle; (**d**) primary follicle; (**e**) secondary follicle.

**Figure 8:**
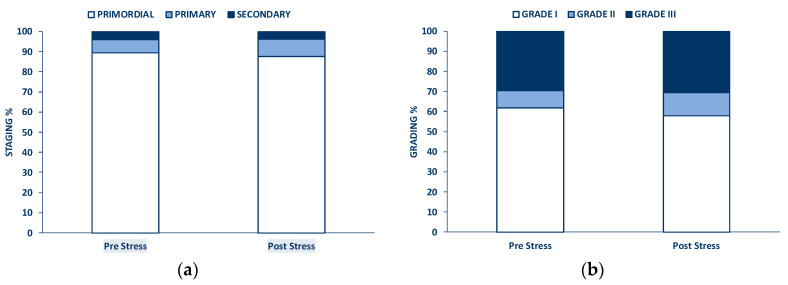
Histological analysis of bovine cortical ovarian tissue before and after mechanical testing: (**a**) staging of follicle growth; (**b**) grading of follicle quality.

Figure 7 shows that the histological analysis of fresh bovine ovarian cortical tissue evidenced its normal appearance (Figure 7a) and the presence of viable stromal cells (Figure 7b), many primordial (Figure 7c), some primary (Figure 7d), and occasional secondary (Figure 7e) follicles. In fresh untested tissue, about 89.4% follicles were primordial, 6.6% were primary, and less than 4% were secondary (Figure 8a). Figure 8b shows that 62% of all follicles were of good quality (grade I) and 30% of poor quality (grade III).

**Figure 9:**
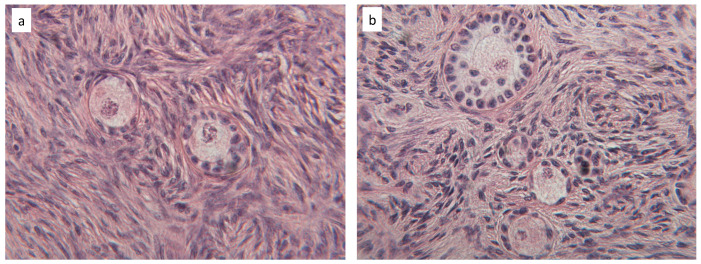
Exemplary histological sections of cortical ovarian tissue before (**a**) and after (**b**) mechanical testing.

Histological analysis of tissue sections before and after performing the mechanical tests did not evidence significant morphological differences, as shown in Figure 8 and Figure 9.

The count of the total follicle number (*n* = 4327) in exemplary fragments (15 pre-testing and 15 post-testing) did not evidence significant differences prior to and after mechanical testing (*n* = 2351 before, and *n* = 1976 after testing, respectively). Figure 8 shows that neither follicle staging nor follicle grading were significantly affected by the mechanical challenge.

## 4. Discussion

As noted above, the ovaries are small almond-shaped structures where oogenesis occurs and where hormones are produced (e.g., oestrogen and progesterone), which are essential for female sexual characteristics and early pregnancy [16]. Their structure is complex and has a distinct polarity from the outer surface to the inner medulla, in which four regions may be distinguished [25]. The ovary surface is lined with a single layer of cells, called the germinal epithelium. A thick connective tissue capsule is present under the epithelium called the tunica albuginea. The tunica is sparsely populated with cells, surrounded by a stroma very rich in collagen. The ovarian cortex is located under the tunica. Its stroma contains high concentrations of collagen, similar to the tunica, but has a much greater density of stromal cells. It houses many primordial and primary follicles, and a few secondary and small antral follicles. The innermost ovarian layer is called the medulla, which is formed by loose connective tissue, is vascularized, and houses large antral follicles. As for many soft tissues, the extracellular matrix contains collagen, glucosaminoglycans (GAGs), and hyaluronans (HAs). It should be noted that it is often hard to distinguish the various regions in histological sections. The thickness and structure of each region is also extremely variable, even in different zones of a given ovarian fragment. Moreover, during each reproductive cycle, the ovaries undergo important structural and functional tissue remodeling, regulated by biochemical and mechanical signals.

The importance of mechanical signaling for ovarian folliculogenesis has been known for about fifteen years. In 2006, Woodruff and colleagues showed that the in vitro culture of murine follicles in stiff matrices would maintain follicle dormancy, whereas less rigid matrices would produce larger, more hormonally productive follicles, featuring higher fertilization rates [17,26]. In 2008, Du et al. [27] reported that treatment at high hydrostatic pressure of porcine oocytes, matured in vitro prior to vitrification, would significantly increase their blastocyst formation rate. In the following years, research efforts have mainly focused on the optimization of culture media, supplements, nutrients, and the amount of oxygen supplied to tissue during in vitro culture; mainly, in static batch culture systems, to grow meiotically competent oocytes, starting from follicles at varying developmental stages [12,14,28]. Only recently, approaches have been proposed that exploit the responsiveness of ovarian tissue to mechanical signals for reproductive purposes. Kawamura and colleagues [18] have reported that reinforcing the disruption of the Hippo signaling pathway in fragmented cortical ovarian tissue with treatment with PI3K stimulators and PTEN inhibitors (both of which activate primordial follicles) allowed for the obtainment of healthy offspring in POI patients. Telfer and McLaughlin [20] have provided anectodical evidence that stretching fragmented ovarian tissue in one direction by more than 10% of the initial length prior to the in vitro culture, enhances primordial follicle activation to the secondary stage. In both approaches, the lack of knowledge of the biomechanical behavior of the cortical ovarian tissue, and the poor quantitative characterization of the mechanical challenge, does not permit correctly estimating the extent of mechanical stimulation and the possible damages to tissue, and ultimately hampers reproducible and effective treatments.

To bridge this gap, in this study we aimed at characterizing the biomechanical behavior of bovine ovarian cortical tissue as a model of human tissue. To the best of our knowledge, this is the first report on the characterization of the biomechanical properties of ovarian cortical tissue. In the absence of information on loads and frequencies used for other studies on ovarian tissue, mechanical tests were performed under conditions similar to those exhibited by, or used for, other reproductive tissues. Hence, tissue was challenged with loads similar to the stresses reported for mouse uterine tissue [29], and at frequencies similar to those used for the mechanical testing of human uterine [30] and cervical [31] tissue. The investigated range of frequencies was centered at about 20 Hz because bovine follicles have been shown to exhibit a frequency-dependent contractile response mediated by norepinephrine release and alpha-adrenergic receptors, with a maximum at about 16 Hz [32]. Tissue was subjected to a short-term mechanical challenge in the low-stress range, not to exceed the 10% maximal fragment deformation, which is considered the ultimate tensile strain for soft tissues, such as tendons and ligaments [33]. 

Dynamic mechanical testing of each tissue fragment yielded a dependence on the test frequency, of both the elastic and the loss modulus, consistent with other reproductive or soft tissues. Figure 3 shows that, similar to most soft tissues, *E_e_* increased with the test frequency and approached an asymptotic value at high frequencies. Similar to that reported for the mechanical behavior of human cervical tissue when characterized by rheometry [31], *E_l_* did not appear to depend on the test frequency in the range investigated in this study. It must be noted that, when the same tissue is characterized in dynamic compression experiments, *E_l_* has been reported to slightly increase with increasing frequencies, in the same range as in this study [34]. For cervical tissue, such a difference may be attributed to different testing and tissue handling procedures. It may also be speculated that for both ovarian and cervical tissue the different behavior reported in [34] may stem from the fact that the mechanical properties of such tissues may also be determined by the interplay between the compressive resistance of GAGs and the tensile resistance of collagen, which may cause an asymmetric tissue response to tension and compression challenges [35].

Figure 4 and Figure 6 show that, as expected, tissue behavior significantly varied from ovary to ovary, possibly as a result of varying nourishment, age, and reproductive development of the bovines. Nonetheless, fragment deformation never exceeded 7%, even at the highest average applied stress, as shown in Figure 5. Tensile dynamic tests and creep tests showed that tissue consistently exhibited a non-linear viscoelastic behavior (Figure 3, Figure 5, and Figure 6). This is further confirmed by the agreement between experimental creep data and predictions of the viscous model (Figure 5). The values of the loss tangent, *tan δ*, consistently lower than unity and the initial slope of the deformation vs. time creep curves, suggest that, under the investigated conditions, tissue exhibited a dominant elastic character (Table 1 and Figure 3). This is consistent with the composition of the cortical stroma, largely made of collagen (i.e., 0.34 g_collagen_/g_dry weight_) [36]. It may be speculated that the viscous response shown in Figure 3 was caused by the presence in the stroma of a matrix made of GAGs (0.026 g_collagen_/g_dry weight_) and HAs (4 × 10^−5^ g_collagen_/g_dry weight_) that embeds collagen fibers and fibrils, and by the sliding of such fibers and fibrils, with respect to the matrix favored by the high hydration state of the stroma [36]. As was expected, the tissue elastic modulus *E_c_* increased, and the loss tangent *tan δ* decreased with increasing average applied stress at given frequency (Table 1). The characterization technique yielded sound values of the tissue elastic modulus which were in the low range of those reported for reproductive tissues subjected in nature to more relevant mechanical challenges than the ovary, such as vaginal tissue (i.e., ca. 0.95–25 MPa), and pelvic ligaments, such as the cardinal ligament (i.e., ca. 0.5–7 MPa), the uterosacral ligament (i.e., ca. 0.75–30 MPa), and the round ligament (ca. 0.7–14 MPa) [37].

In the course of the creep experiments at higher average applied stresses the tissue fragment exhibited some irreversible deformation, and in one case the fragment ruptured. This suggests that to avoid damage, care should be exerted when ovarian cortical tissue is challenged, even at the low stress and strain used for this study. However, the histological analysis of tissue before and after the mechanical tests showed that tissue structure did not suffer significant damage under any condition. In fact, Figure 8 shows, for an exemplary fragment, that there was never evidence of tissue tear. Moreover, the fact that follicles number, staging, and grading (Figure 7) did not change significantly after testing suggests that the mechanical challenges used for the study did not cause follicle expulsion, nor evident rupture of ovarian cells.

A limitation to the study is that a thorough mechanical characterization of such a complex tissue as the ovary would require challenging the tissue along different directions. As noted above, and to the best of our knowledge, this was the first study to investigate the mechanical behavior of ovarian tissue, and we aimed at gathering preliminary information that would allow the planning of further, more comprehensive studies. However, it must be noted that setting up more comprehensive studies is not easy, nor straightforward. In fact, some important practical limitations would have to be overcome. It is very difficult to reproducibly orient and cut tissue fragments so that they may be tested along a reference direction; the small size of the ovaries compared to the size of fragments required for mechanical testing, and the greatly varying properties of the ovarian wall, with the position in the ovaries, make it very difficult (if possible at all) to obtain flat fragments, with uniform wall properties; last but not least, the structural organization of the ovary unpredictably and greatly varies from one individual to the other.

That reported should be considered as a preliminary study towards the development of bioreactors in which ovarian tissue is challenged with both mechanical and biological signals, as a means to guiding follicle activation, and even maturation, for in vitro reproduction purposes. The study permitted identifying mechanical challenges that in the short-term do not cause significant damage to ovarian tissue structure and cells. Next, future studies will aim at researching longer-term mechanical challenges (e.g., loads, frequencies, and duration of the challenge), and in vitro culture conditions that reproducibly enhance, in controlled fashion, follicle activation and maturation, as compared to conventional culture, without causing harm to tissue.

## 5. Conclusions

In this study, we characterized the response of fragments of bovine ovarian cortical tissue to short-term dynamic mechanical tests. The tissue exhibited a nonlinear viscoelastic behavior, with elastic modulus and loss tangent consistent with the high collagen content of the stroma, and within the range of the mechanical properties reported for other reproductive tissues. To the best of our knowledge, this is the first reported mechanical characterization of ovarian cortical tissue. The study also permitted identifying mechanical challenges that do not cause significant damage to ovarian tissue structure and cells, as assessed by histology. Such information could be useful to design bioreactors in which mechanical cues are used, together with suitable biochemical signals, to guide follicle activation, and even maturation, for in vitro reproduction purposes. It is important to notice the large ovary-to-ovary variation in tissue properties. For application purposes, this calls for more extensive characterization work on a larger sample of tissue, over a greater stress range, and for longer periods of mechanical testing, comparable to the time tissue should be cultured in vitro to promote follicle activation and maturation.

## Figures and Tables

**Table 1:** Mechanical properties of cortical ovarian tissue.

Average Stress *σ_m_* [kPa]	Average *E_e_* [MPa]	*tan δ* [-]	Creep Rate [%/s^−1^]
3.75	1.20 ± 0.72	0.34 ± 0.52	0.72 ± 0.17
7.50	1.49 ± 0.74	0.42 ± 0.44	
12.50	1.90 ± 1.02	0.21 ± 0.26	
